# Bacterial Skin Infections in Livestock and Plant-Based Alternatives to Their Antibiotic Treatment

**DOI:** 10.3390/ani11082473

**Published:** 2021-08-23

**Authors:** Lucie Mala, Klara Lalouckova, Eva Skrivanova

**Affiliations:** 1 Department of Microbiology, Nutrition and Dietetics, Faculty of Agrobiology, Food and Natural Resources, Czech University of Life Sciences Prague, Kamycka 129, 165 00 Prague, Czech Republic; malalucie@af.czu.cz (L.M.); lalouckova@af.czu.cz (K.L.); 2 Department of Nutritional Physiology and Animal Product Quality, Institute of Animal Science, Pratelstvi 815, 104 00 Prague, Czech Republic

**Keywords:** wounds, *Staphylococcus aureus*, antibiotic resistance, phytochemicals

## Abstract

**Simple Summary:**

Bacterial skin infections in livestock are among the factors promoting antibiotic use. The use of antimicrobial agents has been shown to contribute to the increased prevalence of resistant bacterial strains. The rapid emergence and spread of resistant bacteria are a worldwide problem. With regard to the development of bacterial antibiotic resistance, phytochemicals are considered as possible substitutions of antimicrobial agents. In the field of plant-derived extracts, a number of studies deserve review because of the severity of the effects of resistant species of bacteria. This review presents current knowledge of plant-derived compounds, focusing on their modes of antibacterial action against pathogenic bacteria causing skin infections in livestock. Finally, great attention is given to specific plants that have antibacterial effects and are used in the healing and wound treatment of farm animals.

**Abstract:**

Due to its large surface area, the skin is susceptible to various injuries, possibly accompanied by the entrance of infective agents into the body. Commensal organisms that constitute the skin microbiota play important roles in the orchestration of cutaneous homeostasis and immune competence. The opportunistic pathogen *Staphylococcus aureus* is present as part of the normal biota of the skin and mucous membranes in both humans and animals, but can cause disease when it invades the body either due to trauma or because of the impaired immune response of the host. Colonization of livestock skin by *S. aureus* is a precursor for majority of bacterial skin infections, which range from boils to sepsis, with the best-characterized being bovine mastitis. Antibiotic treatment of these infections can contribute to the promotion of resistant bacterial strains and even to multidrug resistance. The development of antibiotic resistance to currently available antibiotics is a worldwide problem. Considering the increasing ability of bacteria to effectively resist antibacterial agents, it is important to reduce the livestock consumption of antibiotics to preserve antibiotic effectiveness in the future. Plants are recognized as sources of various bioactive substances, including antibacterial activity towards clinically important microorganisms. This review provides an overview of the current knowledge on the major groups of phytochemicals with antibacterial activity and their modes of action. It also provides a list of currently known and used plant species aimed at treating or preventing bacterial skin infections in livestock.

## 1. Introduction

The skin is the largest organ of animal and human bodies, is the outermost and first line of defense against infectious agents and is easily exposed to physical and chemical agents and different pathogens that cause a wide variety of infections and wounds [1]. Among its functions is the ability to protect the internal body from mechanical impacts and pressure, restriction of the influence of temperature changes, lowering the potential impact of microorganisms, limiting radiation effects and preventing the entrance of different chemicals into the body [2].

The skin is an ecosystem composed of diverse habitats with an abundance of folds, invaginations and specialized niches that support a wide range of microorganisms [3]. The microbiota of the skin is dependent on the specific body site and includes bacteria (e.g., *Proteobacteria*, *Corynebacterium* and *Staphylococcus* spp.), fungi (e.g., *Malassezia*) and viruses (e.g., *Capripox*) [1]. These microorganisms play important roles in the host defense against pathogens and in the development of the host immune system [1,4,5]. A healthy skin microbiota contributes to host fitness by occupying pathogen adhesion sites and producing pathogen inhibitors [1]. *Staphylococcus aureus* is a common pathogen that can cause both localized and systemic infections. Some bacteria, including *Staphylococcus epidermis* and *Corynebacterium* spp., can inhibit or reverse the growth of *S. aureus* [6]. Competitive interactions between beneficial and pathogenic skin microbes may, therefore, play roles in preventing disease in livestock [7]. In contrast to humans, the skin of livestock and other animals is mostly covered with dense fur or feathers. Thus, the microbial composition differs among species [8]. Body location, biological sex, age, geographic location, diet, captivity versus living in the wild, proximity to other animals, maternal transfer and disease states are also important influences on the microbial community structure in animals [9]. Previous studies have described the microbial composition in different skin regions, with *Staphylococcus* spp. and *Corynebacterium* spp. predominating in most areas, *Propionibacterium* spp. predominating in sebaceous areas and gram-negative (G−) organisms (such as Betaproteobacteria) colonizing dry skin areas, such as the forearm and leg [8,10,11,12].

In veterinary medicine, skin infections are common clinical issues. Farm animals can be injured in several ways, e.g., during transport, being kicked or bitten by another animal or by striking against hard objects [13]. Breaches in the skin can lead to skin and soft tissue infections, such as folliculitis and furunculosis, and to life-threatening septicemia [14]. As described in Table 1, dermal wounds can be colonized by a mixture of aerobic (*Staphylococcus* spp. and *Streptococcus* spp.) and anaerobic (*Corynebacterium* spp. and *Trueperella* spp.) bacteria [15]. However, the vast majority of bacterial skin infections in animals involve the genus *Staphylococcus* [14].

In chronic wounds, some bacteria, such as *Staphylococcus* spp., *Streptococcus* spp. and *Corynebacterium* spp., are able to form biofilms, defined as a community of bacteria that have irreversibly attached themselves to a biotic or abiotic surface and secrete extracellular polymeric substances, resulting in higher antibiotic resistance and prevention of phagocytosis [16,17]. In most cases, a concentration of antibiotics in excess of 1000-fold is required to kill planktonic bacteria to destroy bacterial biofilms [16]. Biofilm organisms produce extracellular polymeric substances that facilitate attachment and matrix formation and encapsulate the entire assemblage, resulting in alterations in the phenotypes of the organisms and protection from antibiotics and the host immune system [18]. Furthermore, biofilm bacteria demonstrate a decreased growth rate, leaving them in a permanent growth state that is less susceptible to most antibiotics, which are typically designed to target rapidly dividing bacteria [16]. In veterinary and human medicine, the evolution of antibiotic-resistant genes and their spread among bacterial pathogens have important clinical significance [19].

In addition, antibiotic resistance becomes even more complicated with multidrug-resistant bacteria, such as methicillin-resistant *S. aureus* (MRSA) [20]. Livestock have been identified as an emerging reservoir for the transmission of livestock-associated MRSA (LA-MRSA) to humans. LA-MRSA in humans was first detected in 2005 in a reservoir belonging to the CC398 lineage found in pigs and cattle [21]. Direct contact with live pigs is a known risk factor for LA-MRSA infection, and farm workers and veterinarians are more likely to be exposed and have a significantly elevated risk of becoming LA-MRSA carriers [22]. However, transmission of LA-MRSA is not restricted to persons in direct contact with infected animals, as members of their households show a higher level of LA-MRSA carriage compared to the general community [23]. Although CC398 is the main lineage associated with MRSA isolated from livestock, other clonal complexes and sequence types (STs) that are not within the CC398 variant have also been associated with livestock and animal products, including CC9 and CC130, which are both *S. aureus* clones commonly identified in animals [24]. In addition, other studies have reported the presence of LA-MRSA hybrids, specifically CC9/CC398, CC22, CC30, CC705, ST398 and ST425 hybrids. These isolates have been reported in dairy cows, cattle, pigs and poultry and in animal products [25,26,27,28].

Microbial resistance to antibiotics is constantly growing in both human and veterinary medical settings [29]. The aforementioned reasons clearly call for the development of alternatives to antibiotics. Among the studies on various antibiotic alternatives, the exploration of antibacterial properties of phytochemicals has resulted in successful outcomes. An overview of specific phytochemicals with antimicrobial effects and their relevance are described further in the following paragraphs.

## 2. *Staphylococcus aureus* in Livestock

Generally, staphylococci constitute a major group of bacteria inhabiting the skin, skin glands and mucous membranes of humans, other mammals and birds. *Staphylococcus* is a genus of gram-positive (G+) cocci-shaped bacteria in the family Staphylococcaceae that includes both coagulase-positive and coagulase-negative staphylococci [30]. Coagulase is an enzyme typically produced by *S. aureus* that causes blood clotting in the host, and its production has been accepted as the primary criterion for differentiating pathogenic species of staphylococci from commensal strains [31]. Coagulase-positive *Staphylococcus* species are clinically the most important opportunistic pathogens in many animals and can be considered a potential source of infection and dissemination to the environment [32].

*S. aureus* is an opportunistic pathogen of several animal species and was first recognized as an etiological agent more than 130 years ago. Animals with persistently colonized body sites (udder and teat skin, muzzle and vagina) represent the primary reservoirs of *S. aureus* and sources of infection for other animals and humans [14]. Overall, the ancient development of agriculture and the recent industrialization and globalization of the livestock industry have contributed to increased opportunities for the cross-species transfer of bacteria, and this spread has probably had a profound effect on the emergence of pathogens. The potential for transmission of *S. aureus* from animals to humans and vice versa is well known [33,34]. Transmission usually occurs by direct contact, often via the hands, with colonized or infected animals or people or with contaminated equipment and surfaces. The most common transmission pathways include the transfer from an infected mammary gland to an uninfected gland via fomites, such as milking equipment, or via the milker´s hands, by uncontrolled animal trafficking between different farms and by handling or eating food contaminated with *S. aureus* [35]. Strict hygiene at the time of milking, segregation of any livestock with *S. aureus* infection and intensive culling of those infected might be required to reduce the prevalence and incidence of highly transmissible strains of the bacterium [36]. Thus, *S. aureus* has a huge impact on animal health and welfare and causes major economic losses in livestock production [34,37].

*S. aureus* is one of the three major pathogenic *Staphylococcus* species of animal skin, together with *S. hyicus* and *S. intermedius* [38]. *S. aureus* is found in healthy carriers and can induce a broad array of infections ranging from superficial skin diseases to deep infections and septicemia. Virtually any species of warm-blooded animal can be a healthy carrier or can be infected by *S. aureus* with the known manifestations of the infection [39]. Staphylococcal infections, particularly those caused by *S. aureus*, in farm animal species have been studied for many years, particularly with reference to bovine mastitis, which is defined as “inflammation of the mammary gland” [40]. The physical origins of infection have been identified as contagious, environmental or temporal, e.g., occurring during the dry period or lactation [41]. In addition to *S. aureus*, the coliform bacterium *Escherichia coli* and several streptococcal species, including *Streptococcus uberis* and *Streptococcus agalactiae*, are other major causes of mastitis [42]. *E. coli* is almost always taken up from an environmental source, while other pathogens, such as *S. aureus* and *Str. agalactiae*, are typically transmissible; in contrast, *Str. uberis* can usually act in both contagious and environmental forms. In this case, the crucial step of the identification is the diagnosis of the clinical mastitis and its patterns. Using such an approach may ensure appropriate and effective management interventions for the control of the disease at the herd level [43].

Folliculitis, furunculosis and impetigo are other common staphylococcal infections of the skin in livestock, especially in cattle, goats and sheep. Folliculitis- and furunculosis-associated livestock skin infections may be manifested by skin lesions in almost any location on the body [44]. In contrast to impetigo, where the hair follicle is not involved, the early lesions caused by staphylococci are follicular papules, which develop into a transient pustule with hairs emerging through the lesion. With progression, the condition may include focal crusting, leading to alopecia [14]. Another example of *Staphylococcus* spp.-related skin disease in livestock is inflammation of the lower part of the foot in poultry and bumblefoot. These manifestations are often caused by injuries that allow contamination of the subcutaneous tissue in the footpad [45]. All of the aforementioned skin diseases are associated with the pathogenic bacterium *S. aureus* [14].

## 3. Antibiotic Treatment of Bacterial Skin Infections in Livestock

To improve and maintain animal health, antibiotics are applied in many cases. Decreasing the rate of bacterial infections influences not only animal health, but it also improves animal welfare and food safety, respectively [46]. Nevertheless, the use of antibiotics in animal production contributes to the increase of the burden of antimicrobial resistance in a global context [47]. Livestock is exposed to significant quantities of antibiotics, which also leads to their role as reservoirs of bacterial antimicrobial resistance genes. Moreover, animals can be a source of their transmission to humans via the food chain, direct contact and the environment [46].

**Figure 1:**
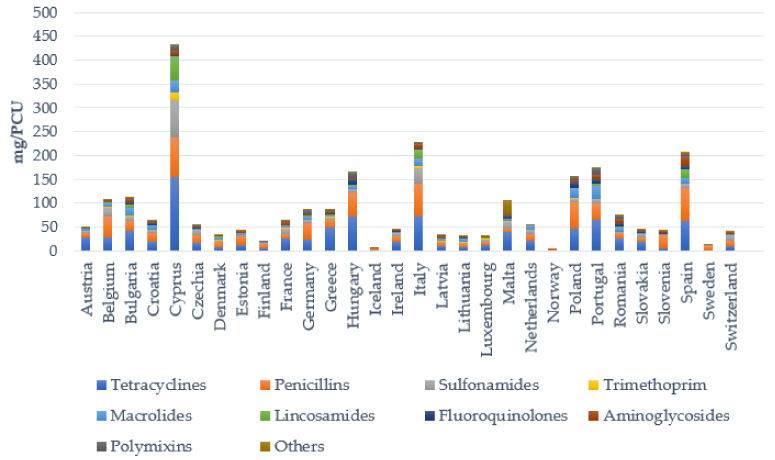
Sales of the various antimicrobial classes in mg/PCU for use in livestock in 30 European countries in 2018.

Antibiotics are extensively used in livestock production systems worldwide (Figure 1) for disease treatment and in some countries for nontherapeutic purposes, such as growth promotion and disease prevention [48]. However, in 2006, the European Union (EU) banned the use of antibiotics for growth-promoting purposes. One of the main reasons for the adoption of the ban was the spread of antibiotic-resistant bacteria to humans [49]. In the EU, antimicrobial usage for therapeutic purposes is particularly high in intensively farmed species such as pigs and poultry and less in extensively farmed cattle (dairy cows are the exceptions) and sheep. In livestock, tetracyclines and penicillins are the most commonly used antimicrobial agents. In European countries, 31.7 mg/PCU (Population Correction Unit) of tetracyclines and 29.7 mg/PCU of penicillins were prescribed for use in veterinary medicine in 2018. The consumption of these substances was up to 30-fold higher than the consumption of other classes of antibiotics [48].

In livestock, tetracyclines have been widely used for many decades to treat a variety of bacterial skin infections, including to treat multidrug-resistant pathogens such as MRSA [50]. Chlortetracycline and oxytetracycline are widely and successfully used for bovine pneumonia prophylaxis and treatment of calf and piglet scours, foot rot, metritis and acute mastitis, as well as for *Pasteurella multocida* infections in poultry [51].

β-Lactams, especially penicillins, are used to treat various conditions, including bovine mastitis, pneumonia in calves, metritis in cows and erysipelas in pigs [51,52]. Penicillin and its derivatives, including methicillin, have been used for the treatment of infections caused by *S. aureus* [53]. However, certain strains of *S. aureus* have developed resistance to these agents and are known as the important veterinary and zoonotic pathogen MRSA. These bacteria show drug resistance to a large number of antibiotics, such as methicillin, penicillin and macrolide antibiotics [20].

In addition to tetracyclines and β-lactams, frequently used antibacterial agent categories for the treatment of bacterial skin diseases in livestock are macrolides, aminoglycosides and fluoroquinolones, in addition to specific substances, e.g., florfenicol [52,54].

### Resistance of Staphylococcal Species to Antibiotics

During recent decades, the continuing rapid development of bacterial resistance to antibiotics has emerged as a major global public health concern, especially with respect to *S. aureus* infection [55]. Bacterial antibiotic resistance occurs naturally over time, usually due to genetic modifications acquired through mutation and selection and/or gene acquisition between strains and species [56]. Resistance to antibiotics that are structurally similar to substances produced by soil bacteria and fungi is likely to have developed long before the clinical use of antibiotic agents in human and veterinary medicine [57]. The selective pressure imposed by the use of antimicrobial agents plays a key role in the emergence of resistant bacteria. Whenever a mixed bacterial population is exposed to antimicrobial agents, it is likely that certain bacteria in the population are resistant to the respective drugs at the concentrations applied. Under selective pressure, the number of these bacteria increases, and some may pass their resistance genes to other members of the population [56]. Generally, bacterial resistance can be classified as clinical and microbiological and, in turn, can be primary (intrinsic) or secondary (acquired). Bacteria can show intrinsic resistance as a result of their own structural characteristics, but can also acquire resistance *via* mutations of chromosomal genes and by horizontal gene transfer, i.e., the lateral movement of genetic information between organisms [58].

**Figure 2:**
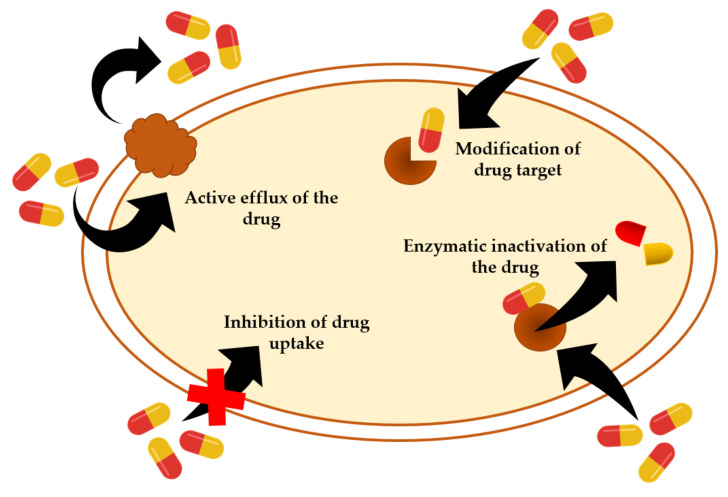
Antimicrobial resistant mechanisms of *Staphylococcus aureus*.

The development of antibiotic resistance by *S. aureus* was first reported in the mid-1940s when a strain developed resistance to penicillin during the production of a hydrolyzing enzyme called penicillinase [59]. *S. aureus* is an adaptable organism with the ability to evolve and become resistant to a wide array of antibiotics [60], and as an opportunistic pathogen, *S. aureus* develops resistance to antimicrobials through different mechanisms. As described Figure 2, these mechanisms include limiting uptake of the drug, modification of the drug target, enzymatic inactivation of the drug and active efflux of the drug [61].

Depending on the antimicrobial involved, the bacteria may use one or several of the aforementioned resistance mechanisms. In particular, the localization of resistance genes on transferable genetic elements such as plasmids and transposons facilitate horizontal transfer of resistance between bacteria [61]. In general, *S. aureus* strains contain a relatively large variety of mobile genetic elements, including plasmids, transposons, bacteriophages, pathogenicity islands and staphylococcal cassette chromosomes. Plasmids and staphylococcal cassette chromosomes in particular have played central roles in conferring resistance to β-lactam antibiotics and vancomycin [62]. In addition, resistance of *S. aureus* is enhanced by the ability of the bacterium to form biofilms that provide protection from the host immune system and antibiotics. Additionally, bacteria in a biofilm state display increased resistance to a stress conditions compared to those in the planktonic state [63]. Growth of the bacteria in a biofilm play an important role during infection by providing a defense against several clearance mechanisms. Specifically, the biofilm matrix can impede the access of certain types of immune defenses, such as macrophages, which display incomplete penetration into the biofilm matrix and phagocytosis [64]. One suggested mechanism for this phenomenon is that the biofilm matrix blocks access to actively growing cells within the biofilm by decreasing antibiotic diffusion rates [65]. Moreover, *S. aureus* biofilms also play an important role in the progression of chronic diseases. Individual cells can disperse from the original biofilm and either seed new sights of infection or mediate an acute infection such as sepsis [66].

Staphylococcal infections have been usually treated with β-lactam antibiotics, but as MRSA strains have spread worldwide, different antibiotics, including vancomycin, daptomycin and linezolid, have been used. Nevertheless, antibiotic MRSA treatment may be challenged by the resistance to oxazolidinone (namely linezolid) and vancomycin [67]. Although the most commonly recommended drug for the treatment of MRSA infections is the glycopeptide vancomycin [68], in some *S. aureus* strains, vancomycin treatment failure started to be prevalent [69]. Three categories of *S. aureus* that are resistant to vancomycin and which have emerged in different locations of the world include vancomycin-intermediate *S. aureus* (VISA), heterogeneous vancomycin-intermediate *S. aureus* (hVISA) and vancomycin-resistant *S. aureus* (VRSA) [70]. Previously, in vitro studies suggested the existence of various mechanisms for vancomycin resistance in MRSA, with the most prevalent one being the decreased permeability and the increased thickness of the cell wall, and hence, a decreased availability of vancomycin for intracellular target molecules. Another type of resistance is caused by plasmid-mediated vancomycin resistance genes, namely *vanA*, *vanB*, *vanD*, *vanE*, *vanF* and *vanG*, which are assumed to be transferred to *S. aureus* from enterococcal species [71,72]. However, vancomycin is not regularly used for the treatment of the diseased animals. In veterinary medicine, there are only few reports of vancomycin-resistant *S. aureus* [73]. 

## 4. Alternatives to Conventional Antibiotics Used to Treat Bacterial Skin Infections in Animals

The problem of antibiotic resistance has been known for decades but has accelerated in recent years [55]. The increase in prevalence of worldwide bacterial resistance to antibiotics requires the development of new antibacterial alternatives. First of all, biosecurity is the most important for preventing the spread of several contagious livestock diseases [74]. In addition, there are a number of antibacterial and immunity boosting compounds used in livestock, such as probiotics and prebiotics [75], synbiotics [76], organic acids [75] and clay minerals [77], but also feed enzymes and phytogenic additives [78]. Antimicrobial peptides, vaccines or bacteriophages can also be used for prevention or treatment of various invasive diseases [79]. Regarding bacterial skin infections in animals, phage therapy is commonly used in bovine mastitis [80] and in chronic wounds of swine [81]. Moreover, there are alternatives to antibiotics with topical use in the form of immunomodulators, which are already used in relation to prevent bovine mastitis [82], or in the form of metal-based antimicrobials, such as copper or zinc. However, in relation to heavy metals, further studies are needed to consider possible toxicity to the animal organism [83].

In traditional medicine, whole plants or mixtures of plants are used rather than isolated compounds [84]. Regarding high antimicrobial activity, pure drugs that are industrially produced or isolated from plants may be chosen for the treatment of bacterial diseases. However, their disadvantage is the lower degree of activity compared to the crude extracts at the comparable concentrations or dose of the active component [85]. Moreover, pure drugs are often more expensive to produce and distribute, and thus, they are often unavailable and/or unaffordable to some parts of the population [86]. In the case of crude extracts, these are complex of hundreds or even thousands of individual constituents which may have increased antibacterial activity due to synergistic action, or conversely lower activity due to the antagonistic action, of the substances with one another [85]. Therefore, research is increasingly focused on the antibacterial potential of these substances [87].

Nature is undoubtedly the richest source of molecules with the most varied biological features. Due to the biodiversity not only between animal and plant kingdoms but also among various species, nature represents the largest library of compounds that has ever existed [88]. Evidence of the use of plants for medicinal purposes dates as far back as 60,000 years ago in both Western and Eastern cultures [89]. Plants serve as useful sources of antimicrobial drugs and offer potential compounds for the development of new antibacterial agents [90]. Regarding antimicrobial action, promising compounds extracted from plants include alkaloids, polyphenols, saponins, tannins, terpenoids, glucosinolates and sulfides [91,92]. Moreover, lectins, together with polyacetylenes, can have the same antimicrobial activity as the aforementioned substances [93].

### Phytochemicals

Phytochemicals are biologically active, plant-derived chemical substances that were traditionally used for medicinal purposes, giving them great therapeutic potential [94]. In ethnoveterinary medicine, parts of plants, such as roots, bark, stems, leaves, flowers and seeds, are used in the form of infusions, decoctions, ointments, powders and drops [95]. Phytochemicals have a wide activity range, according to the associated plant species, topography and climate of the country of origin and may exert different types of effects [94]. The beneficial activity can be attributed to the content of saponins, terpenoids, phenolic compounds, alkaloids and carotenoids in the plant. In addition to their antioxidant, anthelmintic or other beneficial actions, phytochemicals may also have negative impacts, such as hepatotoxic and neurotoxic effects [96]. The exact classification of phytochemicals has not been performed thus far because of their wide variety. In recent years, phytochemicals have been classified as primary or secondary constituents, depending on their roles in plant metabolism [95]. Primary constituents include common sugars, amino acids, proteins and purines and pyrimidines of nucleic acids. Secondary constituents are the remaining plant chemicals, such as alkaloids, terpenes, flavonoids, plant steroids, lignans, saponins, phenolics and glucosides [97]. The basic classification of phytochemicals, together with their main effects, is summarized in Table 2.

Contrary to the beneficial effects, an adverse event when using phytochemicals can be their toxicity. Both effective and toxic concentrations can be almost equal, subsequently interfering with the use of such a substance. In both human and veterinary medicine, an emphasis must be given to toxicity levels testing to prevent the use of lethal or sublethal doses [99].

#### Phytochemicals with Antimicrobial Effects

##### Alkaloids

Many definitions of alkaloids can be found in the literature, but generally, all include the statement that alkaloids are naturally occurring, low-molecular-weight organic substances with nitrogen-containing bases, most often produced by plants [100,101]. Although there is no fundamental taxonomic classification, alkaloids are usually classified based on their chemical structure, biochemical activity or natural origin [102]. From a biosynthetic point of view, alkaloids are derived from amino acids, such as phenylalanine, tyrosine, tryptophan, ornithine and lysine [103]. Based on their origin, three main categories exist: true-, proto- and pseudoalkaloids [102]. With regard to the biogenesis of alkaloids, they can be categorized into different classes according to their precursor (e.g., the largest group of alkaloids, indole alkaloids, are derived from tryptophan), thereby encompassing more than 20 different classes, such as pyrrolizidine alkaloids, tropane alkaloids, pyridine alkaloids, piperidine alkaloids, quinolizidine alkaloids, steroidal alkaloids and others [101,103].

Many alkaloids have important medical uses, whereas others are very toxic to both humans and animals [100]. Alkaloids usually have various potent biological activities and have a bitter taste [101]. Plants from the Papaveraceae and Berberidaceae families produce alkaloids with potential beneficial effects on wound healing. Both plant families produce isoquinoline alkaloids, which exert a range of biochemical effects relevant for medical use, such as alleviation of pain, inhibition of cancer cell growth and inhibition of bacterial growth [104]. To date, different studies have investigated the antimicrobial activity of some Papaveraceae species. The extracts of four annual poppy species showed strong antibacterial activity against the pathogen *S. aureus* [105]. The same study suggested that *Papaver rhoeas* extracts exhibited high inhibitory effects towards *S. aureus*, *E. coli*, *Pseudomonas aeruginosa*, *Salmonella abony* and *Candida albicans* pathogens [106]. In another study, strong antimicrobial activity of extracts from alkaloids in the Papaveraceae family was observed against both G+ and G− bacteria [107,108]. Table 3 lists selected alkaloids and their minimum inhibitory concentrations (MICs) against bacteria.

According to the Clinical and Laboratory Standards Institute (CLSI) [117], penicillin, a β-lactam antibiotic, shows MICs similar to alkaloids against G+ bacteria, specifically *S. aureus* ATCC 29213 (MIC 0.25–2 µg/mL) and *Enterococcus faecalis* ATCC 29212 (MIC 1–4 µg/mL). However, some antimicrobial agents show lower MICs against these bacterial species, primarily towards *S. aureus*. These antibiotics include clindamycin and fusidic acid (both with a MIC of 0.06–0.25 µg/mL). The MICs of these conventional antibiotics against G− pathogens are not included in CLSI tables. Based on the MIC values presented in Table 3, it is obvious that alkaloids show higher antibacterial activity against G+ bacteria than against G− bacteria. This can be explained by the fact that the outer membrane of G− bacteria disables the penetration of numerous antibiotics, and the periplasmic space contains enzymes that can degrade exogenous molecules [118].

Generally, antibacterial mechanisms of action are not equal within alkaloid classes. Synthetic quinolone alkaloids may have respiratory inhibition effects, while isoquinolines, such as berberine, protoberberine, sanguinarine and benzophenanthridine, inhibit cell division by perturbing the Z-ring. Ungeremine, a phenanthridine isoquinoline alkaloid, acts by blocking nucleic acid synthesis. Finally, pergularinine and tylophorinidine, which are indolizidine alkaloids, inhibit nucleic acid synthesis by targeting dihydrofolate reductase [119].

Antibacterial activity is also influenced by structural relationships. Iwasa et al. [120] examined quaternary protoberberine alkaloids and revealed that growth inhibitory activity was influenced more by the type of oxygen substituents on rings A, C and D and particularly by the position of the oxygen functional groups on the ring. Azimi et al. [115] also observed similar results, and four tested alkaloids showed potent antibacterial activity towards *Brucella abortus*. However, jatrorrhizine and columbamine, which have a free hydroxyl group on C-2 or C-3, showed stronger activity than berberine and palmatine, which have no free hydroxyl groups.

With regard to the antibacterial activity, synergistic and antagonistic effects of alkaloids in combination with antimicrobial drugs were observed. Sanguinarine, a benzophenanthridine alkaloid, has strong antibacterial activity against G+ bacteria. In addition, this agent shows synergistic activity with streptomycin and a chelating agent, ethylenediaminetetraacetic acid (EDTA). Tong et al. [121] found a synergistic effect of berberine with β-lactam antibiotics against MRSA.

In contrast to their benefits, some alkaloids may have a toxic effect on both the animal and human body. In livestock, many species of lupines contain quinolizidine or piperidine alkaloids known to possess toxic or teratogenic effect [122]. Norditerpenoid alkaloids act as antagonists blocking the ligand binding sites of nicotinic acetylcholine receptor and causing acute toxicosis in adult animals that can result in death. Additionally, steroid alkaloids from *Veratrum californicum* such as cyclopamine inhibit the hedgehog signaling pathway, which disrupts embryonic development, causing developmental defects [123].

#### Polyphenols

Polyphenols are secondary metabolites ubiquitously distributed in all higher plants. The chemical structures of polyphenols comprise a wide variety of molecules and are generally classified into flavonoids and nonflavonoids [124]. Flavonoids constitute the largest class of polyphenolic compounds, with more than 4,000 structurally unique flavonoids already identified in various plant sources, such as vegetables, fruits and plant-derived beverages (e.g., tea and wine) [125]. Depending on the oxidation state of the central pyran ring, flavonoids can themselves be subdivided into many subclasses: flavonols, flavones, flavanones, anthocyanidins, flavanols and isoflavones. Nonflavonoids include simple phenols, phenolic acids, phenolic amino acids, curcuminoids, stilbenes, lignans and hydrolysable gallotannins and ellagitannins [124].

Generally, polyphenols have strong antibacterial activity against G+ (e.g., *S. aureus*, *Streptococcus mutans*, *Clostridium perfringens*) and G− (e.g., *E. coli*) bacteria, probably due to different mechanisms of action, among which the most convincing identified involves the aggregatory effect on all bacterial cells [124,126]. However, nonflavonoids show weaker antimicrobial activity than flavonoids [127]. Table 4 lists the selected flavonoids and nonflavonoids and their MICs against bacteria.

With regard to the values of the MICs of flavonoids and nonflavonoids in Table 4, the majority of these compounds were found to be more active against pathogenic bacteria, such as *S. aureus*, *Listeria monocytogenes* and *E. coli*, than conventional antibiotics, such as aminoglycosides (range of MICs 0.12–1 µg/mL) and tetracyclines (range of MICs 0.12–4 µg/mL) [117,127]. For this reason, they seem to be a suitable natural alternative towards bacterial pathogens. As reported above, flavonoids are more effective compared to nonflavonoids. This can be explained by the basic structural difference between these classes. Specifically, nonflavonoids contain only one phenol ring and flavonoids contain two phenol rings. Furthermore, the two phenol rings of flavonoids are connected via an oxygen-containing central pyran ring [133].

In phenolic compounds, multiple mechanisms of antibacterial activity have been described. One of these activities involves the interaction of polyphenols with bacterial proteins and cell wall structures [124,126]. The interactions of bacterial cell membranes with active hydroxyl groups of phenolic compounds cause either the disruption of the membrane structure, which induces a loss of cellular content, or the delocalization of electrons, which results in depolarization of bacteria, and thus, affects the proton motive force, reducing the pH gradient across the membrane and the level of the ATP pool [134]. Furthermore, phenolic compounds may inhibit nucleic acid synthesis or cell wall synthesis. The -OH groups of phenolic compounds interact with the cell membrane of bacteria by hydrogen bonding. Importantly, the presence of −OH functional groups is relevant to the antibacterial activity of many phenolics [135]. Moreover, longer aliphatic chain causes stronger hydrophobic properties of the compound, and can possibly enhance the antibacterial effect by enabling the substance to better interact with cytoplasmic membrane [136].

On the other hand, research on the anti-biofilm activity of plant phenolic compounds has revealed that in addition to their destructive effect on bacteria, their “softer” activity leads to biofilm suppression by affecting bacterial regulatory mechanisms, such as quorum sensing and/or other global regulatory systems, without exerting an effect on bacterial growth [137]. Recently published studies have confirmed the anti-biofilm activities of phenolic compounds, especially flavonoids, phenolic acids and tannins. The effects of these phenolic compounds were observed against both G+ and G- biofilm-forming bacteria [138,139,140]. For example, the flavonoids apigenin and quercetin have broad anti-biofilm activity against *S. aureus*, *E. coli* and *Str. mutans* [138,141]. Coumarins, esculin, psoralen and nodakenetin have the same effect on *P. aeruginosa* [142]. Inhibition of biofilm formation by phenolic compounds may be crucial in future applications that prevent medical device biofilm-associated infections both in animals and humans [139].

The synergistic effect of polyphenols in combination with conventional antimicrobial agents against clinical multidrug-resistant bacteria was observed in most studies. The in vitro effect of two flavonols, especially kaempferol and quercetin, in combination with rifampicin (a complex macrocyclic antibiotic), was demonstrated towards MRSA isolates. Regarding the mechanism of action, use of these phytochemicals alone showed slight β-lactamase inhibition, but when combined with rifampicin, the complex exhibited good β-lactamase inhibitory effects [143]. Tea polyphenols have also been particularly proven to synergistically enhance the antimicrobial activity of antimicrobial agents used against MRSA [144].

However, the potential toxicity of some polyphenols in higher concentrations was also reported, such as catechin to damage DNA in mice spleen cells [145]. Moreover, notable negative effects were observed in fibroblast and keratinocyte cell lines after exposure to a high concentration of epicatechin for 24 h or longer [146]. The studies indicate that the positive effects could be obtained from polyphenols in a safe concentration range only [147].

#### Tannins

“Tannin” is a general descriptive name for a group of water-soluble polymeric phenolic substances, which differ from most other natural phenolic compounds in their ability to precipitate proteins such as gelatine from solution [148]. Tannins are found in abundance in tree bark, wood, fruit pods, leaves and roots and in plant galls. Tannins are subclassified into broad groups — proanthocyanidins (condensed tannins, CTs) and gallotannins and ellagitannins (hydrolysable tannins, HTs) [149]. HTs are generally multiple esters of gallic acid with glucose. Structurally, CTs are complexes of oligomers and polymers of flavonoid units (i.e., flavan-3-ols, flavan-3,4-diols and biflavans) linked by carbon-carbon bonds [150]. HTs are found primarily in fruit pods and plant galls, and in contrast to CTs, their degradation products are absorbed by the small intestine of animals and are potentially toxic to ruminants [151].

Tannins inhibit the growth of diverse microbes, such as bacteria, fungi and yeasts [148]. Generally, the antimicrobial activity of tannins against G+ bacteria has been reported to be greater than that against G− bacteria [152]. Table 5 shows a list of selected tannins and their MICs against bacteria.

Based on the MIC values presented in Table 5, it is obvious that tannins and the abovementioned alkaloids have a greater antibacterial effect towards G+ bacteria and a lesser antibacterial effect towards G– bacterial species. This pattern corresponds with facts previously mentioned in the subsection on alkaloids [118]. Because of the low MICs of selected tannins, such as tannic acid, tannins can be used as alternatives to antibiotics against pathogenic bacteria.

The mechanisms proposed thus far to explain tannin antimicrobial activity include inhibition of extracellular microbial enzymes, deprivation of the substrates required for microbial growth, direct action on microbial metabolism through inhibition of oxidative phosphorylation, metal ion deprivation or the formation of complexes with the cell membrane of bacteria causing morphological changes in the cell wall and increasing membrane permeability [148].

Gallotannins show higher antibacterial effects than ellagitannins. Tannic acid consisting of a central glucose and 10 galloyl groups had the lowest MIC value and is the most studied tannic acid with a broad spectrum of antibacterial activities [160]. Compared to proanthocyanidins, HTs show less pronounced antibacterial activities. Hydroxylation at positions 5 and 7 on the B ring plays an important role in the antimicrobial effect of flavonols [161].

Tannins have been reported to show anti-biofilm effects. For example, tannic acid inhibited biofilm formation in the pathogenic bacteria *S. aureus* and *E. coli* [162,163]. In *S. aureus*, the anti-biofilm effect of tannic acid was mainly associated with the production of the protein “immunodominant staphylococcal antigen A”, which is a putative lytic transglycosylase that can cleave β-1,4-glycosidic bonds between the amino sugars of the bacterial cell wall, N-acetylmuramic acid and N-acetylglucosamine. Cleavage of the peptidoglycan layer can lead to a reduction in *S. aureus* biofilm thickness [164].

Although tannins are widely used due to their beneficial properties, the negative effects of tannins are also known. Tannin-containing plant can be less palatable due to the binding of tannin to salivary glycoproteins, resulting in an unpleasant taste [165]. Tannins also exhibit antinutritional properties by forming complexes with minor elements (such as calcium, magnesium or phosphorus), as well as with major elements (such as carbohydrates and proteins), rendering them unavailable for the utilization by the body. They may also form complexes with enzymes. High concentrations of tannins (up to 5.0 g/100 g dry matter) may be toxic to the animal by causing irritation and desquamation of the intestinal mucosa, kidneys and lives lesions, ulcers and even death [166].

#### Essential Oils

As concentrated hydrophobic liquids, essential oils (EOs) are complex mixtures of volatile compounds produced by living organisms and isolated exclusively by physical means (pressing and distillation) from a whole plant or plant part of known taxonomic origin [167]. According to their chemical structure, these oils are alcohols, ethers or oxides, aldehydes, ketones, esters, amines, amides, phenols, heterocycles and mostly terpenes [168]. EOs and their constituents are known for their bioactive properties, including their antiseptic or antibacterial action [135,169,170]. Currently, the use of these phytochemicals to protect livestock from infections, mainly on organic farms, is becoming a common practice [171].

The activity of EOs is not equal, as they differ in their chemical structure. Some can also show synergisms [135]. The antimicrobial effect of EOs and their constituents has been explored in many in vitro assays, with variable results. The effect is highly dependent on the type of the compound, as well as on the bacterial type. Notably, G− bacteria are more tolerant to the action of essential oils than G+ bacteria [172,173]. This finding can be attributed to the different structure of G− bacterial outer membrane and its limiting diffusion ability of hydrophobic compounds [174]. Moreover, the lipophilic ends of lipoteichoic acid in the cell membrane of G+ bacteria may ease the infiltration of hydrophobic EO compounds [175]. An important attribute of EOs is their hydrophobic character, which can interfere with the membrane permeability [174]. This can then cause changes in ion channels (Na^+^, K^+^, Ca^2+^ or Cl^−^) and subsequent leakage of ions and other cellular molecules [175]. Even though a certain amount of leakage from bacterial cells can be tolerated without loss of viability, this phenomenon has its limitations. After the greater loss of cellular contents or critical output of molecules and ions, cell death is inevitable [176].

Generally, the chemical structure of EOs affects their antibacterial mode of action. EOs characterized by a high level of hydroxyl groups (i.e., thymol, eugenol, carvacrol and terpineol) are strongly reactive. This can lead to the interference with bacterial enzymes and their inactivation [177]. These compounds have high antimicrobial activity against pathogenic bacteria such as *E. coli* and *Salmonella typhimurium*, both of which are potential risk factors for enteric infections in livestock, especially in poultry. Moreover, the bacteria *S. aureus*, *Bacillus cereus*, *L. monocytogenes*, *Campylobacter jejuni*, *Lactobacillus sake* and *Helicobacter pylori* are susceptible to these EOs [98,178].

Some studies have concluded that whole EOs have greater antibacterial activity than mixtures consisting of major EO components [179,180]. This suggests that minor components in EOs are somewhat needed for EO inhibitory effect. They can have a synergistic effect or a potentiating influence [168]. Additive and synergistic effects of 1,8-cineole and aromadendrene against MRSA, vancomycin-resistant enterococci and *E. faecalis* have been reported [181]. Furthermore, Lambert et al. [169] observed an additive antimicrobial effect of carvacrol and thymol against *S. aureus* and *P. aeruginosa*. Oregano oil with gentamicin showed synergistic effects against *S. aureus* and *Bacillus subtilis* and *B. cereus* [182]. However, there are many EO components that have not been tested for determining their potential to enhance the efficacy of antibiotics [183].

Various studies have shown the toxicity of EOs in vitro and in vivo. EOs are commonly associated with hepatotoxicity, nephrotoxicity, changes in the blood vessels and oxidative stress that occur as a result of acute intoxication [184]. For example, a study focusing on the toxicity of an EO obtained from *Syzygium aromaticum* (containing mainly eugenol and caryophyllene) confirmed its negative effect on rats after intraperitoneal injection (0.125 mg/kg), resulting in changes of kidney tissue [185]. Some EOs have also been associated with toxicity to reproductive system. A recent study in rats demonstrated the prenatal toxicity of *Verbena officinalis* EO. Embryo-fetotoxicity was observed as evidence of the decrease in fetal weight, tail length and head cranium [186].

#### Terpenoids

Terpenoids, also referred to as terpenes, are the main constituents of EOs and constitute the largest group of natural compounds, accounting for more than 40,000 individual substances [187]. A majority of these compounds are abundant in flowers, fruits and vegetables. In particular, terpenes can be found at high concentrations in the reproductive structures and foliage of plants throughout and immediately following flowering [188]. Chemically, terpenes are usually cyclic unsaturated hydrocarbons with different degrees of oxygen in the constituent groups attached to the basic isoprene skeleton. The nomenclature of terpenoids depends on the number of isoprene structures and carbon atoms in the molecule; therefore, they are commonly classified as monoterpenes (C_10_), sesquiterpenes (C_15_), diterpenes (C_20_), triterpenes (C_30_), tetraterpenes (C_40_) and polyterpenes (C_>40_) [189].

Terpenoids have several biological functions in higher plants. They are the key components of membrane structures, function as photosynthetic pigments and contain phytohormones (abscisic acid and gibberellins), and terpenoids known as ubiquinones are involved in mitochondrial electron transport [190]. Plant oils that included terpenes in their composition showed promising in vivo bactericidal activity, especially against various G+ and G− pathogenic bacteria [191,192]. Table 6 lists selected terpenoids and their MICs against bacteria.

The MICs presented in Table 6 demonstrate that terpenoids are active against both G+ and G– bacteria. However, polyterpenoids, particularly nimbolide, show an MIC of 8 µg/mL. This MIC indicates that some G+ bacteria are resistant to the effects of certain terpenoids. This resistance may be caused by a thick layer of peptidoglycan in the cell wall, which confers the cells with rigidity, making the passage of antimicrobial agents difficult [194].

Due to their lipophilic character, terpenes easily permeate through the cell wall and cell membrane. Disruption of membrane integrity and potential, leakage of cellular contents, denaturation of cytoplasmic proteins and inactivation of cellular enzymes lead to bacterial cell death [191]. Terpenoids also play key roles in the clinical industry [192].

#### Saponins

Saponins constitute a diverse group of bioorganic compounds that are widely distributed in the plant kingdom with a rigid skeleton of at least four hydrocarbon rings to which sugars in groups of one or two are attached [195]. Saponin phytochemical molecules consist of two key moieties, a hydrophilic sugar moiety and a lipophilic sapogenin moiety, the combination of which contributes to the characteristic soapy/detergent nature of saponins [196]. Based on the different types of sapogenin, there are two classes of saponins. One class consists of steroidal saponins, in which sapogenin contains the characteristic four-ringed steroid nucleus, with typical extra furan and pyran heterocyclic rings. Due to the lack of the extra rings, the ginsenosides in ginseng are considered to be the second class of saponins, known as the triterpenoid saponins, even though they exhibit a steroidal structure [197].

Due to their amphiphilic nature, saponins show a wide range of biological activities, such as cytotoxic, anticancer, insecticidal, molluscicidal, anti-inflammatory, antifungal, antiviral and antibacterial activities [195,198]. Regarding antimicrobial activity, this group of phytochemicals inhibits the growth of G+ and G− bacteria and yeasts and molds. For example, saponins from *Yucca* exhibit antimicrobial activity against G+ cells but do not act on G− bacteria [199]. However, *Salvia officinalis* extracts showed antibacterial effects against the G- pathogen *E. coli*. [200,201]. Table 7 lists selected saponins and their MICs against bacteria.

The antibacterial mechanism of saponins against bacteria is not completely understood [207]. In general, the effect of saponins against bacteria is often weak [208]. It seems that saponins can interact with the bacterial outer membrane, increasing its permeability. There are inconsistent reports on the activity of glycosidic and aglycone forms of saponins. It was shown that bacterial enzymes could decrease the antibacterial effect of saponins through the hydrolysis of sugar chains [207]. Avato et al. [209] reported that the aglycone component of saponins had antibacterial activities and that the sugar chains are not critical for this activity, whereas Khan et al. [210] proved that the presence of sugar chains is important for the biological effects of the extracts. However, research conducted with *E. coli* demonstrated that oleanolic acid (OA), a known triterpenoid saponin, can also moderately affect efflux pumps, which can directly interfere with the viability of this bacterial species [211]. Other mechanisms of action of OA may be associated with the induction of a stress response [212]. Kurek et al. [213] verified that triterpenoid saponins inhibit peptidoglycan turnover in *L. monocytogenes*, affecting the amount of muropeptides and, ultimately, the cellular wall of bacteria, suggesting that this biochemical pathway can be a target for both triterpenes.

Regarding the antimicrobial effects, saponins also possess a significant antifungal activity. The mechanism of action involves pore formation and loss of membrane integrity. The mode of action is similar to the hemolytic activity of saponins [214]. A mechanism of action for the triterpene saponin of oats, (avenacin) was proposed. Briefly, it includes the administration of the aglycone fragments into the cell membrane, followed by binding to sterols. The next step comprises the interaction of sugar residues and the formation of sterol-saponin complexes. These complexes can lead to the restructuring of membrane lipids, the formation of pores and cell lysis [215].

Many studies have also investigated the synergistic effect resulting from the combination of antibiotics with saponins, discovering new ways to treat infectious diseases [216,217]. For example, a synergistic effect was observed for the glycoside of OA in combination with a tetracycline antibiotic against *S. aureus* and *E. coli* [217].

Negative effects of saponins are also known. Some of these substances have the ability to disrupt erythrocytes due to their interactions with the sterols [195]. For instance, the study of Yoshikawa et al. [218] confirmed the acute and sub-chronic toxicity of *Camellia sasanqua* seed saponins in mice. Tested mice showed severe gastrointestinal tract distension and submucosal changes in the small intestine, indicating that the toxic target could be the gastrointestinal system.

#### Organosulfur Compounds

Alliaceous vegetables are considered to be enriched within a large variety of beneficial substances, such as sulfur-containing compounds [219]. Organosulfur compounds are defined as organic molecules containing one or more carbon–sulfur bonds. These compounds are particularly prominent in the Alliaceae and Brassicaceae families of plants [220]. Organosulfur compounds in *Allium* and *Brassica* plants are called thiosulfinates and glucosinolates. These groups of phytochemicals are converted to various new sulfur-containing materials that exhibit a variety of bioactive properties via a number of biosynthetic reactions [221].

Thiosulfonates (TSFs) are the most studied compounds among the active constituents of *Allium* vegetables. Allicin, or diallyl thiosulfinate, is the main active substance of garlic (*Allium sativum* L., *Amaryllidaceae*). Injury to the tissue of the garlic wedge leads to the release of the enzyme alliinase, which produces allicin from the basic compound alliin [222]. Alliinase is characterized by both carbon and sulfur stereochemistry, although it occurs naturally as a racemate [223]. Allicin has broad low-level antimicrobial activity against G+ and G− bacteria, including against antibiotic-resistant bacterial strains and fungi [224].

Glucosinolates (GLSs) constitute a class of organic compounds that are formed from glucose and an amino acid and contain sulfur and nitrogen. Rich sources of these compounds are *Brassica* vegetables [225]. GLSs are present in plants with the enzyme myrosinase, which hydrolyses GLSs into active compounds, such as isothiocyanates, after tissue disruption. Isothiocyanates are the main active substances of GLSs and have strong antibacterial effects against both gram-positive and gram-negative bacteria [226].

Generally, organosulfur compounds—allicin, benzyl isothiocyanate, propyl-propane thiosulfonate and ajoene—showed high in vitro antibacterial activity. Allicin is the only compound from this group that was tested in animal models and clinical trials for the treatment of bacterial infections [227,228,229]. Table 8 lists the selected organosulfur compounds and their MICs against bacteria.

TSFs, including allicin, use their –S(O)-S- group to inhibit bacterial growth. The group generally reacts with the −SH group of cellular proteins to generate mixed disulfides. The inhibitory action of TSFs is inactivated by sulfhydryl compounds such as cysteine [234]. All cysteine sulfoxides-derived antibacterial substances in alliums are believed to act via the equal mechanism, as their antimicrobial activities are inactivated by cysteine, except allyl alcohol [235]. The mechanism of allicin antimicrobial activity has been reported, which is due to the inhibition of sulfhydryl-dependent enzymes, including alcohol dehydrogenase, thioredoxin reductase and RNA polymerase [236]. Furthermore, allicin has been found to partially inhibit DNA and protein synthesis. The immediate effect of allicin on RNA has also been proven, which indicates the possibility that RNA is a target of allicin [237].

The mode of antibacterial action of GLSs is probably the inhibition of the activity of thiol groups in key bacterial enzymes or the blockade of electron transport and ATP synthesis [238]. The synergistic effect of TSFs and GLSs in combination with conventional antimicrobial agents against pathogenic bacteria was observed in most studies. For example, allicin shows synergistic and adjuvant activity with antibiotics such as oxacillin and cefazolin against *S. aureus* and *P. aeruginosa* [239]. Allicin-β-lactam combinations offer the promise of clinical utility, especially when synergism is demonstrated by in vivo experimental studies [240]. Additionally, isothiocyanates display synergy with conventional antibiotics. It was demonstrated that 2-(4-hydroxyphenyl)ethyl isothiocyanate had antimicrobial synergism with aminoglycosides such as streptomycin against *E. coli* and *S. aureus* [241]. However, small changes in the concentrations of both isothiocyanate and streptomycin affected their combined action, changing from synergism to the suppression of antimicrobial activity [242].

If organosulfur compounds are applied in a high concentration (up to 200 mg/kg of the body weight), an imbalance between chemical stress and response capacity leads to contradictory results along with adverse side effects, such as toxicity to the heart, brain, liver and other organs [243]. In the study of Alnaqeeb et al. [244], the intraperitoneal administration of garlic extract, in both low (50 mg/kg of the body weight) and high doses (500 mg/kg of the body weight), caused damage to the lung and liver in rats.

## 5. Plants with Antibacterial and Wound Healing Effects Used in Livestock (In Vitro Studies)

Many diseases affect livestock and other animals, and causal organisms of diseases include bacteria, viruses, protozoa, fungi and helminth parasites [245]. Plant phytochemical remedies are used for many livestock animals, including ovines, bovines, swine, poultry and rabbits. Prime disorders addressed by these substances comprise wounds and dermatological complications as well as gastrointestinal disorders and postnatal maladies [246]. It is essential to evaluate not only the bioactivity but also the safety of plant treatments when their use is to be promoted and potentially developed for commercial purposes. In vivo experiments are expensive and ethically complex; hence, many studies involve in vitro investigations of a particular bioactivity [245]. A number of in vitro experiments have been performed, in which plants were used for treating livestock bacterial skin diseases and in the healing process. Table 9 contains a list of these plants.

In livestock, the plants most extensively used in the healing process and treatment of skin infections are in the Fabaceae and Asteraceae families.

## 6. Plants with Antibacterial and Wound Healing Effects Used in Livestock (In Vivo Studies)

Compared to in vitro studies, the number of in vivo studies of plant extracts is significantly lower. Current in vivo experiments of plant-derived compounds related to bacterial skin infections involve predominantly testing in rats (Sprague-Dawley and Wistar) [259] or in mice [260,261], and to a lesser extent in rabbits [262]. Bibliographic research highlights a low number of in vivo experiments using livestock when evaluating the possible effect plants extracts in treating bacterial skin diseases. However, the antibacterial action of an herbal spray comprising of *C. deodara*, *Curcuma longa*, *E. globulus* and *G. glabra* has been assessed by Hase et al. [263] for its therapeutic effect on subclinical mastitis in bovines. Treatment by herbal spray achieved a 60% cure rate. Abboud et al. [264] studied the effect of 10%mixture of *L. angustifolia* and *T. vulgaris* in dairy cows, using it as an intramammary infusion or externally. After four days of treatment, it has been discovered that a substantial decrease in the bacterial colony count occurred. The most potent antimicrobial effect was achieved by massaging the udder with a mixture of EOs. In vivo activity of EOs was confirmed also in *O. vulgare* [265]. In addition, in vivo studies on skin diseases caused by mites were previously carried out. Kebede and Negese [266] tested the antimicrobial effect of *E. globulus* EO and *Cymbopogon citratus* EO in goats infected by *Sarcoptes scabiei* var. *caprae*. Animals were topically treated two times for 14 days interval and compared with non-treated and treated (diazone and ivermectin) controls. The infected goats treated with the EOs were cured completely.

## 7. Conclusions

Given the general concerns of the increased prevalence of antimicrobial resistance of microorganisms, especially *S. aureus*, the most common multidrug-resistant bacterium causing skin diseases in animals, there is an effort to limit the use of antibacterial agents to the lowest acceptable level. In livestock, staphylococcal bacterial infections are dangerous not only for their harmful effects on animal health but also for their potential for transmission from animals to humans and vice versa. Staphylococcal infections, therefore, have a huge impact on animal health and welfare and cause major economic losses in livestock production. In this regard, another problem is the treatment of staphylococcal infections, which requires antibiotic therapy. However, the administration of antibiotics to livestock can promote the development and spread of multidrug-resistant bacterial strains. The present review reports that plants are valuable sources of antibacterial compounds. Moreover, the combined therapy of selected phytochemicals with antibiotics can improve their pharmacokinetic and pharmacodynamic properties. Thus, phytochemicals open a wide range of possibilities for new antibacterial therapies in veterinary medicine. However, the use of plant substances may also have adverse effects on animals. The overall effect on the health status of animals may depend a great deal on the chemistry of the compounds, their concentration in the diet and the amount consumed. In this review, most of the cited studies were based on in vitro experiments; therefore, further detailed in vivo experiments are needed.

## Figures and Tables

**Table 1:** The most common bacterial pathogens in the diseased skin of livestock [14].

Species of Livestock	Bacterial Pathogens
Cattle	*Actinomyces bovis*, *Bacteroides melaninogenicus*, *Staphylococcus aureus*, *S. hyicus*, *Streptococcus dysgalactiae*, *Fusobacterium necrophorum*, *Moraxella bovis*, *Trueperella pyogenes*
Pigs	*Dermatophylus congolensis*, *S. hyicus*, *S. intermedius*, *S. chromogenes*, *S. sciuri*
Goats	*Dermatophylus congolensis*, *S. aureus*, *S. hyicus*, *S. haemolyticus*, *S. warneri*, *S. epidermidis*, *S. chromogenes*, *S. caprae*, *S. simulans*
Sheep	*Dermatophylus congolensis*, *Corynebacterium pseudotuberculosis*, *Pithomyces fungus*, *S. aureus*, *S. xylosus*, *S. epidermidis*, *Str. dysgalactiae*
Poultry	*S. aureus*, *S. hyicus*

**Table 2:** Classification of phytochemicals and their main effects.

References	Phytochemicals	Activity
[92]	Alkaloids	Antimicrobial, anti-inflammatory
[91]	Polyphenols	Anticarcinogenic, antimicrobial, antioxidative, antithrombic, immunomodulatory properties, anti-inflammatory, influence on blood pressure
[91]	Saponins	Anticarcinogenic, antimicrobial, immunomodulatory properties, influence on blood pressure
[92]	Tannins	Antimicrobial, anti-inflammatory, antioxidative
[98]	Essential oils	Antimicrobial, anti-inflammatory
[91]	Terpenoids	Anticarcinogenic, antimicrobial, anti-inflammatory, cholesterol-lowering effect
[91]	Carotenoids	Anticarcinogenic, antioxidative, immunomodulatory properties, cholesterol-lowering effect
[91]	Organosulfur compounds	Anticarcinogenic, antimicrobial, antioxidative, antithrombic, immunomodulatory properties, anti-inflammatory, influence on blood pressure
[91]	Phytosterols	Anticarcinogenic, cholesterol-lowering effect
[91]	Protease inhibitors	Anticarcinogenic, antioxidative, modulate blood glucose levels
[91]	Phytoestrogens	Anticarcinogenic, antioxidative, immunomodulatory properties

**Table 3:** The minimum inhibitory concentrations of selected alkaloids against gram-positive and gram-negative bacteria.

References	Alkaloids		MIC (µg/mL)	
	Classes of Alkaloids	Specific Representatives	G+	G−
[109,110,111]	Quinolines alkaloids	4-methyl quinolone, 8-hydroxyquinolone, evocarpine	2–50	8–100
[112,113,114,115]	Isoquinolines, aporphines, phenanthrenes	Lysicamine, artabotrine, liridine, sanguinarine, berberine, jatrorhizine, columbamine, buesgenine, palmitine	0.5–2.5	0.78–32
[116]	Other alkaloids	Carmichaedine	8	-

MIC: minimum inhibitory concentration; G+: gram-positive bacteria; G−: gram-negative bacteria.

**Table 4:** The minimum inhibitory concentrations of selected phenolic compounds against gram-positive and gram-negative bacteria.

References	Phenolic Compounds		MIC (µg/mL)	
	Classes of Phenolics	Specific Representatives	G+	G−
[128,129,130,131,132]	Flavonoids	Quercetin, myricetin, brazilin, neobavaisoflavonde, lupinifolin, 6,8-diprenyleriodictyol, pseudarflavone A	0.5–62.5	4–32
[128,129,130,131,132]	Nonflavonoids	3´-demethoxy-6-O-demethylisoguaiacin, 4-epi-larreatricin, dihydroguaiaretic acid, resveratrol	12.5–>1000	25–1280

MIC: minimum inhibitory concentration; G+: gram-positive bacteria; G−: gram-negative bacteria.

**Table 5:** The minimum inhibitory concentrations of selected tannins against gram-positive and gram-negative bacteria.

References	Tannins		MIC (µg/mL)	
	Classes of Tannins	Specific Representatives	G+	G−
[153,154,155]	Gallotannins	Tannic acid, hexa-*O*-galloylglucose, hepta-O-galloylglucose, 1,2,6-tri-O-galloyl-β-D-glucopyranose	0.16–1000	5–3200
[154,156,157]	Ellagitannins	Punicalagin, corilagin, tellimagrandin I, tercatain, chebulagic acid, isorugosin A, davidiinm castalagin	0.25–1000	4–3200
[154,158,159]	Proanthocyanidins	Procyanidin A1, procyanidin B1, procyanidin B2, Procyanidin B3, procyanidin B4, rhodonidin A, prodelphidin, epicatechin	0.1–100	2–800

MIC: minimum inhibitory concentration; G+: gram-positive bacteria; G−: gram-negative bacteria.

**Table 6:** The minimum inhibitory concentrations of selected terpenoids against gram-positive and gram-negative bacteria.

References	Terpenoids		MIC (µg/mL)	
	Classes of Terpenoids	Specific Representatives	G+	G−
[193]	Monoterpenoids	Carvacrol, thymol, linalool, citronellol, α-terpineol	0.007–32	0.015–55
[193]	Sesquiterpenoids	Xanthorrhizol, onopordopicrin	0.5–86.2	2.2–6.8
[193]	Diterpenoids	Carnosol, carnosic acid, rosmanol, lasiodin, bafoudiosbulbin C, (-)-copalic acid, dehydrobietic acid	0.5–25	3.1–64
[193]	Polyterpenoids	Nimbolide	8	-

MIC: minimum inhibitory concentration; G+: gram-positive bacteria; G−: gram-negative bacteria.

**Table 7:** The minimum inhibitory concentrations of selected saponins against gram-positive and gram-negative bacteria.

References	Saponins		MIC (µg/mL)	
	Classes of Saponins	Specific Representatives	G+	G−
[202,203]	Steroidal saponins	Progenin II, diosgenin, spirosta-5,25(27)-diene-1β,3β-diol-1-*O*-α-l-rhamnopyranosyl- (1→ 2)-β-d-fucopyranoside (fruticoside H)	7.8–>256	128–>256
[204,205,206]	Triterpenoid saponins	Oleanolic acid, betulinic acid, moronic acid, ursolic acid, friedelane-3,11-dione	1.52–64	1–100

MIC: minimum inhibitory concentration; G+: gram-positive bacteria; G−: gram-negative bacteria.

**Table 8:** The minimum inhibitory concentrations of selected organosulfur compounds against gram-positive and gram-negative bacteria.

References	Organosulfur Compounds		MIC (µg/mL)	
	Classes of Organosulfur Compounds	Specific Representatives	G+	G−
[230,231,232]	Thiosulfinates	Ajoene, Z-ajoene, allicin, propyl-propane thiosulfinate	4–20	0.5–>500
[233]	Glucosinolates	Benzyl-isothiocyanate, allyl-isothiocyanate	4–40	10–40

MIC: minimum inhibitory concentration; G+: gram-positive bacteria; G−: gram-negative bacteria.

**Table 9:** List of plants used to treat skin infections in livestock.

References	Family	Botanical Name	Treatment	Part of Plant
[247]	Altingiaceae	*Liquidambar orientalis*	Bovine mastitis	Leaves
[248,249]	Apiaceae	*Eryngium planum, Conium maculatum, Sanicula europaea*	Bovine mastitis, wound healing	Herb
[250]	Arecaceae	*Areca catechu*	Bovine mastitis	Seeds
[251]	Asphodelaceae	*Aloe* species	Bacterial skin diseases, wound healing	Leaves
[252]	Asparagaceae	*Achyranthes aspera, Drimia maritima*	Bovine mastitis, other bacterial skin diseases	Leaves
[248,252,253,254,255,256]	Asteraceae	*Achillea millefolium, Arnica montana, Artemisia nilagirica, Calendula officinalis, Eclipta prostrata, Eupatorium triplinerve, Blumea lacera, Cyanthillium cinereum, Haplocarpha scaposa, Helianthus annuus, Matricaria recutita, Mikania scandens, Saussurea costus, Solidago virgaurea, Stevia rebaudiana, Tagetes erecta, T. patula, Tridax procumbens, Vernonia* species, *Wedelia chinensis*	Bovine mastitis, wound healing	Flowers, leaves, roots
[248]	Boraginaceae	*Bourreria orbicularis, Heliotropium indicum, Symphitum officinale*	Wound healing	Barks, leaves, roots
[256]	Bignoniaceae	*Spathodea campanulate*	Bovine mastitis	Leaves
[253]	Capparaceae	*Capparis zeylanica*	Wound healing	Leaves
[250]	Clusiaceae	*Garcinia mangostana*	Bovine mastitis	Pericarp
[253,257]	Cucurbitaceae	*Coccinia grandis*	Bacterial skin diseases, wound healing	Fruits, leaves, roots
[253,258]	Dilleniaceae	*Dillenia indica*	Bacterial skin diseases, wound healing	Fruits
[253]	Ebenaceae	*Diospyros malabarica*	Wound healing	Leaves
[248,252,253,254,255,256]	Euphorbiaceae	*Acalipha indica, Euphorbia hirta, Croton bonplandianum, C. macrostachyus, Jatropha zeyheri, Ricinus communis*	Wound healing	Leaves, roots
[248]	Fabaceae	*Acacia nilotica, Aeschynomene indica, Butanea monosperma, Calpurnia aurea, Cullen corylifolium, Crocosmia aurea, Glycyrrhiza glabra, Pterocarpus marsupium, Rhynchosia capitate, Saraca indica, Senna alata, S. sophera, S. alexandria, Schotia latifolia, Vigna unguiculata*	Bovine mastitis, other bacterial skin diseases, wound healing	Fruits, leaves
[256]	Hypericaceae	*Hypericum perforatum, H. revolutum*	Wound healing	Flowers, roots
[253]	Chenopodiaceae	*Chenopodium bonus-henricus*	Wound healing	Leaves
[250]	Lamiaceae	*Anisomeles indica, Leucas aspera, Lavandula angustifolia, Mentha, arvensis, Minthostachys verticillata, Ocimum sanctum, Ocimum tenuiflorum, Origanum vulgare, Plectranthus amboinicus, P. ambiguous, Tectona grandis, Thymus vulgaris, Vitex negundo*	Bovine mastitis, other bacterial skin diseases, wound healing	Flowers, leaves
[253,257]	Lauraceae	*Litsea glutinosa*	Wound healing	Barks, leaves
[253,258]	Malvaceae	*Gossypium herbaceium, Malva neglecta, M. sylvestris, Sida cordifolia*	Bacterial skin diseases, wound healing	Herbs, leaves
[253]	Menispermaceae	*Tinospora sinensis*	Wound healing	Stem
[248,252,253,256]	Molluginaceae	*Glinus lotoides*	Wound healing	Latex, leaves
[248]	Moraceae	*Ficus benghalensis, F. caria, F. racemosa, F.thonningi, Morus nigrai*	Bacterial skin diseases, wound healing	Latex, leaves, roots
[256]	Myrtaceae	*Eucalyptus globulus, Syzygium cumini*	Bacterial skin diseases	Leaves
[253]	Papaveraceae	*Fumaria indica, Papaver somniferum, Chelidonium majus*	Bovine mastitis, wound healing	Leaves
[250]	Pandaceae	*Pandanus foetidus*	Wound healing	Leaves
[253,257]	Pinaceae	*Cedrus deodara, Picea abies*	Bovine mastitis, other bacterial skin diseases	Bark
[253,258]	Poaceae	*Bambusa bambos, Cynodon dactylon*	Bovine mastitis, wound healing	Leaves, shoots
[253]	Polygonaceae	*Rumex obtusifolius*	Bacterial skin diseases	Leaves, roots
[248,252,253,254,255]	Rhamnaceae	*Ziziphus mucronata, Z. spina-christi*	Bovine mastitis	Leaves, roots
[248]	Rubiaceae	*Morinda citrifolia*	Bovine mastitis	Leaves
[256]	Salvadoraceae	*Salvadora persica*	Bacterial skin diseases, wound healing	Leaves
[253]	Solanaceae	*Atropa belladonna, Datura metel, Nicotiana tabacum, Solanum hastifolium, S. americanum, S. sodomeum, S. virginianum, Withania somnifera*	Bovine mastitis, other bacterial skin diseases, wound healing	Leaves, roots
[250]	Symplocaceae	*Symplocos racemose*	Bacterial skin diseases	Bark
[253,257]	Vitaceae	*Cissus quandrangularis*	Bacterial skin diseases	Aerial parts

## Data Availability

The data presented in this study are available within the review.
